# Smooth muscle cells-specific loss of OCT4 accelerates neointima formation after acute vascular injury

**DOI:** 10.3389/fcvm.2023.1276945

**Published:** 2023-10-23

**Authors:** Junchul Shin, Svyatoslav Tkachenko, Delphine Gomez, Rupande Tripathi, Gary K. Owens, Olga A. Cherepanova

**Affiliations:** ^1^ Department of Cardiovascular and Metabolic Sciences, Lerner Research Institute, Cleveland Clinic, Cleveland, OH, United States; ^2^ Department of Genetics and Genome Sciences, Case Western Reserve University, Cleveland, OH, United States; ^3^ Department of Medicine, Division of Cardiology, University of Pittsburgh, Pittsburgh, PA, United States; ^4^ Robert M. Berne Cardiovascular Research Center, University of Virginia, Charlottesville, VA, United States

**Keywords:** smooth muscle cells, OCT4, proliferation, differentiation, vascular injury

## Abstract

**Introduction:**

There is growing evidence that smooth muscle cell (**SMC**) phenotypic transitions play critical roles during normal developmental and tissue recovery processes and in pathological conditions such as atherosclerosis. However, the molecular mechanisms responsible for these transitions are not well understood. Recently, we found that the embryonic stem cell/induced pluripotent stem cell (**iPSC**) factor OCT4, which was believed to be silenced in somatic cells, plays an atheroprotective role in SMC, and regulates angiogenesis after corneal alkali burn and hindlimb ischemia by mediating microvascular SMC and pericyte migration. However, the kinetics of OCT4 activation in arterial SMC and its role in acute pathological conditions are still unknown.

**Methods and Results:**

Here, using an *Oct4*-IRES-GFP reporter mouse model, we found that OCT4 is reactivated in the carotid artery 18 hours post-acute ligation-induced injury, a common *in vivo* model of the SMC phenotypic transitions. Next, using a tamoxifen-inducible *Myh11*-CreERT2 *Oct4* knockout mouse model, we found that the loss of OCT4, specifically in SMC, led to accelerated neointima formation and increased tunica media following carotid artery ligation, at least in part by increasing SMC proliferation within the media. Bulk RNA sequencing analysis on the cultured SMC revealed significant down-regulation of the SMC contractile markers and dysregulation of the genes belonging to the regulation of cell proliferation and, positive and negative regulation for cell migration ontological groups following genetic inactivation of *Oct4*. We also found that loss of *Oct4* resulted in suppression of contractile SMC markers after the injury and in cultured aortic SMC. Further mechanistic studies revealed that OCT4 regulates SMC contractile genes, ACTA2 and TAGLN, at least in part by direct binding to the promoters of these genes.

**Conclusion:**

These results demonstrate that the pluripotency factor OCT4 is quickly activated in SMC after the acute vascular injury and inhibits SMC hyperproliferation, which may be protective in preventing excessive neointima formation.

## Introduction

Vascular smooth muscle cells (SMC) are highly specialized cells that regulate blood vessel tone and blood pressure. Mature differentiated SMC have a low cell proliferation and migration rate, low synthetic activity, and express a unique repertoire of contractile proteins, ion channels, and signaling molecules required to support cell contractile functions (1, 2). However, SMC are very plastic and can reversibly change their phenotype in response to environmental cues (3). This process, called SMC phenotypic switching, mimics some aspects of the dedifferentiation process (1). The phenotypically modulated non-contractile SMC has reduced expression of the unique combination of contractile marker proteins, also known as smooth muscle marker genes, that are required for their contractile functions, including SM myosin heavy chain (MYH11), SM α-actin (ACTA2), SM 22α (TAGLN), and calponin (CNN1) (3). The cells stop behaving as differentiated SMCs and revert to a more pluripotent-like state capable of migrating, laying down the extracellular matrix, and proliferating. Phenotypic switching of vascular SMC plays a critical role in restenosis after vascular injury (4) and in atherosclerosis (5) development through a dramatic increase in the rate of cell proliferation, migration, synthesis of extracellular matrix proteins and pro-inflammatory molecules, and a decrease in the expression of SMC-specific marker genes. Proliferation and migration of vascular SMC are normal processes necessary for tissue repair in response to vascular wall damage. However, in some pathological conditions, SMC promote pathological vascular wall remodeling, at least in part through increased migration and proliferation.

Smooth muscle cell phenotypic transitions are regulated via the complex transduction of environmental cue-mediated signals. Platelet-Derived Growth Factors (PDGF) (6, 7), oxidized phospholipids (8), and pro-inflammatory cytokines, such as IL1β (9), play critical roles in SMC phenotypic switching. We previously demonstrated that KLF4, a Krϋppel-like family of transcriptional repressors members, is a master regulator of SMC phenotypic switching *in vitro* and *in vivo* (10, 11). KLF4 is induced after PDGF-BB, oxidized phospholipid, or IL1β exposure, and KLF4-signaling has been shown to attenuate SRF/myocardin binding to SMC CArG boxes (11). In addition, KLF4 has been implicated in the epigenetic reprogramming of SMC (12). Recently, it has been shown that microRNAs (13) and long noncoding RNAs (14) are also involved in regulating SMC differentiation and modulation of SMC phenotype.

Recent SMC-lineage tracing animal studies in combination with single-cell (sc)RNA-sequencing revealed that phenotypically modified SMC demonstrate remarkable phenotypic plasticity in many pathological conditions, including atherosclerosis (15–18), obesity and hyperglycemia (19), and aneurysm formation (20). For example, studies from several groups uncovered that in atherosclerosis, SMC down-regulate contractile markers and obtain markers of other cell origins, including macrophages, osteoblasts, myofibroblasts, and mesenchymal stem cell-like cells, indicating that SMC phenotypic switching is multi-directional. Moreover, multi-color lineage tracing studies revealed that SMC-derived cells within the atherosclerotic lesion (21) or post-vascular injury neointima (22) are oligoclonal, generated from only a few pre-existing SMC, indicating high proliferation activity of these cells (23). However, despite much knowledge about molecular mechanisms of SMC dedifferentiation, little is known about specific mechanisms switching cells to specific phenotypic states.

Recently, we found that the pluripotency factor OCT4, which was believed to be silenced in somatic cells, plays an atheroprotective role in SMC in that genetic inactivation of OCT4 in SMC led to the worsening of atherosclerosis development in atherosclerotic *Apoe*^‒/‒^ mice at least in part by preventing SMC migration and investment into the protective fibrous cap (5). *In vitro*, we demonstrated that OCT4 is reactivated in SMC in response to oxidized phospholipids, hypoxia, and Toll-like receptor 3 activation via hydroxymethylation of the *OCT4* promoter and KLF4- and HIF1⍺-dependent mechanisms (5).

Atherosclerosis is a chronic inflammatory disease that takes a long time to develop. Nevertheless, SMC phenotypic transition may happen very quickly in response to acute vascular injury that leads to a hypoxic environment, indicating that OCT4 can also play a role in SMC phenotypic switching during vascular remodeling after injury. Of particular relevance, we found that OCT4 plays an important role in microvascular SMC and pericytes by regulating angiogenesis following non-atherosclerosis-related injuries, including corneal alkali burn and hind limb ischemia (24). However, it is still unknown if protective SMC-specific OCT4-dependent mechanisms are activated in other acute pathological settings to enhance injury repair and/or vascular remodeling. Also, it is unknown whether OCT4 directly regulates genes involved in the contractile machinery of SMC.

In this study, we utilized a carotid ligation injury, the common *in vivo* model of the SMC phenotypic switching (25), to investigate the role of the SMC-derived OCT4 in acute vascular conditions. We demonstrate that OCT4 is quickly upregulated in the tunica media after injury, and genetic inactivation of OCT4, specifically in SMC, leads to significant increases in the neointima formation post-injury, at least in part via increases in SMC proliferation. Mechanistically, we demonstrate that OCT4 directly binds to the SMC contractile gene promoters (TAGLN and ACTA2), and loss of OCT4 results in quicker suppression of ACTA2 after injury compared to control. Our findings indicate that OCT4 has an important fundamental role in SMC in numerous chronic and acute stress conditions.

## Materials and methods

### Mice

Animal protocols were approved by the University of Virginia and Lerner Research Institute Animal Care and Use Committees. *Oct4^Flox/Flox^* (Pou5f1^tm1Scho^) mice were generously provided by H. Schöler (Max Planck Institute, Germany) and *Myh11-CreERT2* [Tg(Myh11-cre/ERT2)1Soff] mice by S. Offermanns (Max Planck Institute, Germany). Oct4-IRES-GFP mice were purchased from the Jackson Laboratory (B6.129S4-*Pou5f1^tm2Jae^*/J, #008214).

*Oct4^Flox/Flox^* mice were mated with *Myh11-CreERT2* mice to generate *Oct4^Flox/WT^Myh11-CreERT2* mice. The *Myh11-CreERT2* transgene is located on the Y-chromosome, thus only male mice were used for experiments. *Oct4^Flox/WT^;Myh11-CreERT2* males were mated with *Oct4^Flox/WT^* females to generate experimental *Oct4^Flox/Flox^;Myh11-CreERT2* and control *Oct4^WT/WT^;Myh11-CreERT2* male littermate mice. The genotype of conditional *Oct4* mice and *Myh11-CreERT2* mice were identified as described previously (26, 27).

To activate Cre-recombinase, tamoxifen (Sigma-Aldrich, 1 mg/100 µl in peanut oil) was administered through eight daily intraperitoneal injections starting at 5–6 weeks of age.

#### Carotid ligation injury

Carotid artery ligation injury was performed in *Oct4^Δ/Δ^;Myh11-CreERT2*, control *Oct4^WT/WT^;Myh11-CreERT2*, *Oct4-IRES-GFP^+/−^* and control *Oct4-IRES-GFP^−/−^* mice as described previously (25, 28). The right carotid artery was completely sutured proximal to the carotid bifurcation using a sterile suture silk 6-0, permanently occluding blood flow to the superficial and deep branches. The left carotid artery served as an uninjured control. The right (injured) and left (uninjured/control) carotid arteries were harvested 1, 3, or 21 days after injury and fixed with 4% paraformaldehyde. Paraffin-embedded carotids were sectioned at 5 µm thickness from the ligature to the aortic arch. Morphometric and immunohistochemical analyses were conducted using 3–4 sections per artery. The right and left carotid arteries were harvested the same day after injury for RNA extraction. The injured *Oct4-IRES-GFP* mice tissues were harvested 18 h after injury, washed with PBS, and immediately subjected to epi-fluorescence microscopy.

### Smooth muscle cell culture

Mouse aortic SMC were isolated from the thoracic aortas of 6-week-old male C57BL/6 mice or *Oct4^WT/WT^* and *Oct4^Δ/Δ^* mice following tamoxifen injections using the collagenase/elastase protocol, as previously described (5). For experiments with POVPC treatment, confluent SMC were starved with serum-free media (SFM), including DMEM/F12 (Gibco), 100 U/ml penicillin/streptomycin [Gibco], 1.6 mM/L L-glutamine [Gibco], to induce growth arrest and differentiation of cells. After culturing in SFM for 48 h, mouse aortic SMCs were treated with DMSO-vehicle, or POVPC (10 µg/ml; Cayman Chemicals). Cells were used for experiments between passages 5–12.

### Immunohistochemical and morphometric analyses

The morphometric characteristics of the carotids were analyzed using the Modified Russell-Movat (Movat) staining. Immunohistochemistry (IH) was conducted with antibodies for OCT4-biotin (clone C10, Santa-Cruz Biotechnology Inc.), ACTA2-biotin (clone1A4, Santa Cruz Biotechnology Inc.), and Ki67 (ab15580, Abcam). Staining for IH was visualized by DAB (Acros Organics). Images were acquired with Zeiss Axioskope2 fitted with an AxioCamMR3 camera. Image acquisition was performed with AxioVision40 V4.6.3.0 software (Carl Zeiss Imaging Solution). The settings were fixed at the beginning of the acquisition and analysis steps. Vessel morphometry [external elastic lamina (EEL), neointima, and tunica media] and areas of positive immunohistochemical staining were quantified using ImagePro Plus 7.0 software (Media Cybernetics, Inc) as previously described (5, 28, 29). The number of OCT4 positive cells are counted using ImageJ (NIH) software after immunostaining. Supplementary Figure S1 explains the strategy for OCT4 signal analysis.

“Live” Oct4-GFP expression was detected by epi-fluorescence microscopy (Zeiss). ImageJ (NIH) software was used for quantification. The threshold for a positive signal was set by minimal positive pixels in the native vessel pictured in the same field as the injured vessel.

### RNA extraction, cDNA preparation, and quantitative real-time RT-PCR

Total RNA isolation from cultured cells and carotid tissues was performed using Trizol reagent (Invitrogen) following the manufacturer's protocol. Extracted RNA was treated with DNaseI (Invitrogen), and 1 mg of RNA was reverse transcribed with iScript cDNA synthesis kit (BioRad). Real-time RT-PCR was performed on a C1000^™^ Thermal Cycler CFX96^™^ (BioRad) using SensiFAST^™^ SYBR NO-ROX Mix (Bioline) and specific primers for mouse *Myh11, Cnn1, Tagln,* and *18s RNA*, as previously described (5). The gene expression was normalized to *18s RNA*. All experiments were conducted in triplicates or duplicates and performed in 2–3 independent experiments.

### Cell viability assay

Cell viability was measured the CellTiter 96® AQ_ueous_ One Solution Cell Proliferation assay (MTS assay) (Cell Promega) as previously described (5). Experiments were done in triplicates for each experimental group and performed in 2–3 independent experiments.

### Chromatin immunoprecipitation (ChIP) assay

ChIP assays were performed as previously described (5). The sheared chromatin was immunoprecipitated with 2 µg of the OCT4 antibodies (Santa-Cruz, sc-20691) and immune complexes were recovered with magnetic beads (Millipore). Quantitative RT-PCR was performed using SensiFAST^™^ SYBR NO-ROX Mix (Bioline) and primers specific for the *Acta2 and Tagln* promoters. Primers—*Acta2* BS1 F:GGCAACACAGGCTGGTTAAT, R: GCCATTAGCTGAGGACTTGG; BS2 F: TGCGATCTCTGTATTTGAGCA, R:TGGATTCTGGGAGTGCTTCT, *Taglin* BS1 F: CAAGTCCGGGTAACAAGGAA, R: AAGTCTGCTTGGCTCACCAC; BS2 F: TTGGAAAGGCCACTTTGAAC, R: AGAAAGGGGGAGGCATTCTA.

### Statistics

The Kolmogorov-Smirnov test was performed to test for the normality of the data. Two-tailed Student's *t*-tests or one-way ANOVA were used to compare two groups of continuous variables with normal distribution. Multiple-way ANOVA was used for multiple-group comparison. Two-group comparisons with non-normal distributions were analyzed using the non-parametric ANOVA based on the Wilcoxon rank sum test. Fisher's exact test was used for categorical data. *P* < 0.05 was considered significant. GraphPad software 9.4.0 was used for all statistical analyses. All *in vitro* experiments were performed in duplicates or triplicates in 2–5 independent experiments. The numbers of animals used for *in vivo* experiments are indicated in the Figures.

## Results

### The pluripotency factor OCT4 is upregulated after acute carotid ligation injury

Previous studies have detected the pluripotent isoform OCT4 in somatic cells by detecting mRNA or protein levels. However, these methods are controversial due to several potential false positives associated with endogenous OCT4 transcript and protein detection (30). We used several genetic animal models to detect the presence and function of OCT4 in SMC without relying on antibody or gene expression methods.

**Figure 1:**
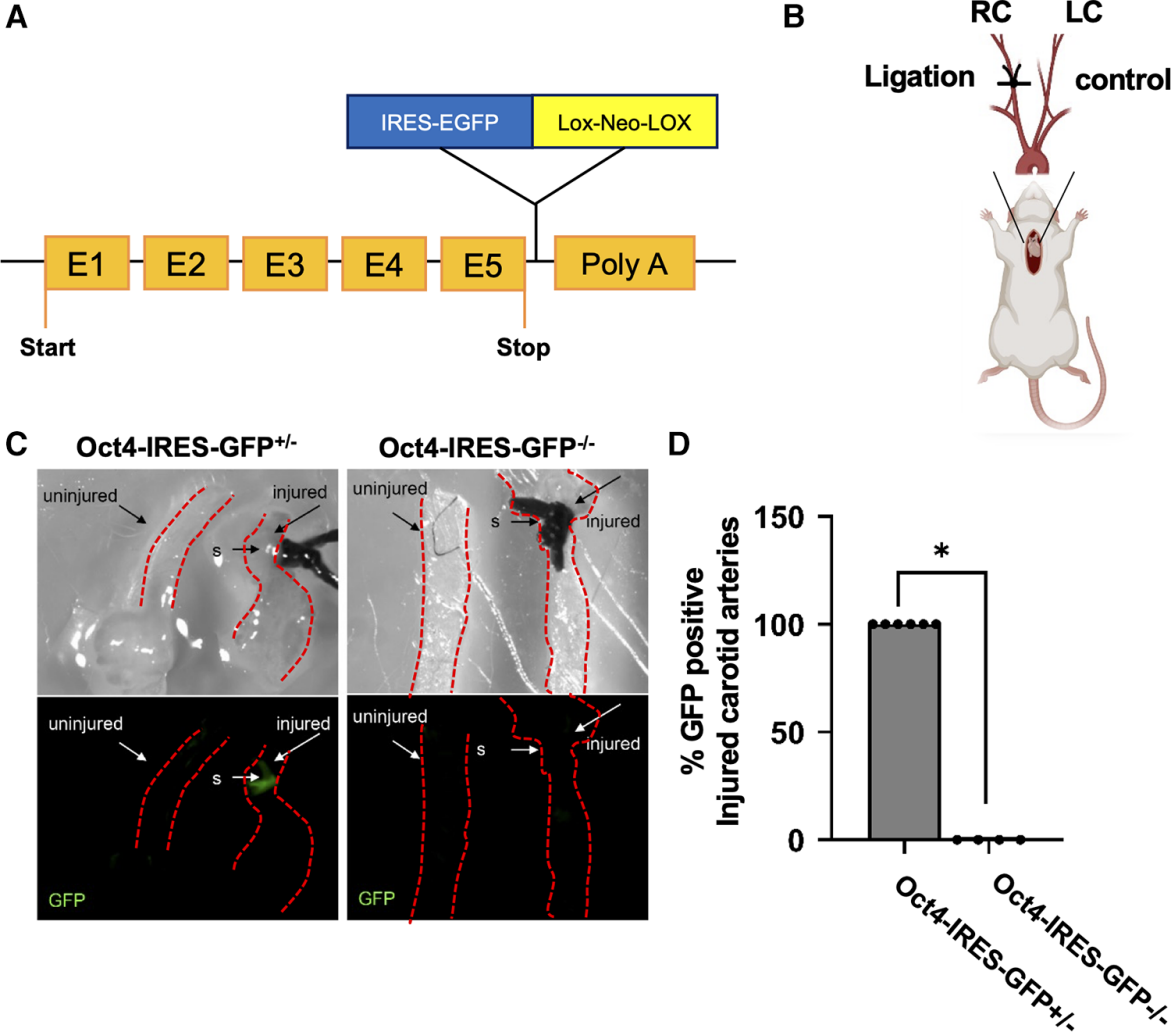
Oct4-IRES-GFP reporter mice showed activation of OCT4-GFP signal after carotid ligation injury. (**A**) Schematic of the *Oct4-IRES-GFP* mouse model. (**B**) Schematic representation for the carotid artery ligation injury model. (**C**) *Oct4-GFP* expression was upregulated in the carotid arteries of Oct4-IRES-GFP^+/–^ mice in response to carotid ligation injury compared to collateral uninjured carotid artery 18 h post-injury. Top panel—bright field, bottom panel—epi-fluorescence illumination of “live” GFP, s-suture. (**D**) Percent *Oct4-GFP*^+^ injured carotid arteries. *P = 0.005, Fisher's exact test *Oct4-IRES-GFP*^+/–^ (*n* = 6) vs. *Oct4-IRES-GFP*^–/–^ (*n* = 4).

*First*, to evaluate the activation of OCT4 after carotid ligation injury, we utilized *Oct4-IRES-GFP* reporter mice, in which the endogenous Oct4 locus controls GFP expression (31) (Figure 1A). Specifically, the right carotid artery was completely ligated proximal to the carotid bifurcation (Figure 1B). The left carotid artery was used as an uninjured control. Importantly, we found that the *Oct4*-GFP signal was activated in the carotid arteries of *Oct4-IRES-GFP*^+/‒^ mice in response to ligation injury as compared to collateral uninjured carotid artery 18 h post-injury (Figures 1C,D).

**Figure 2:**
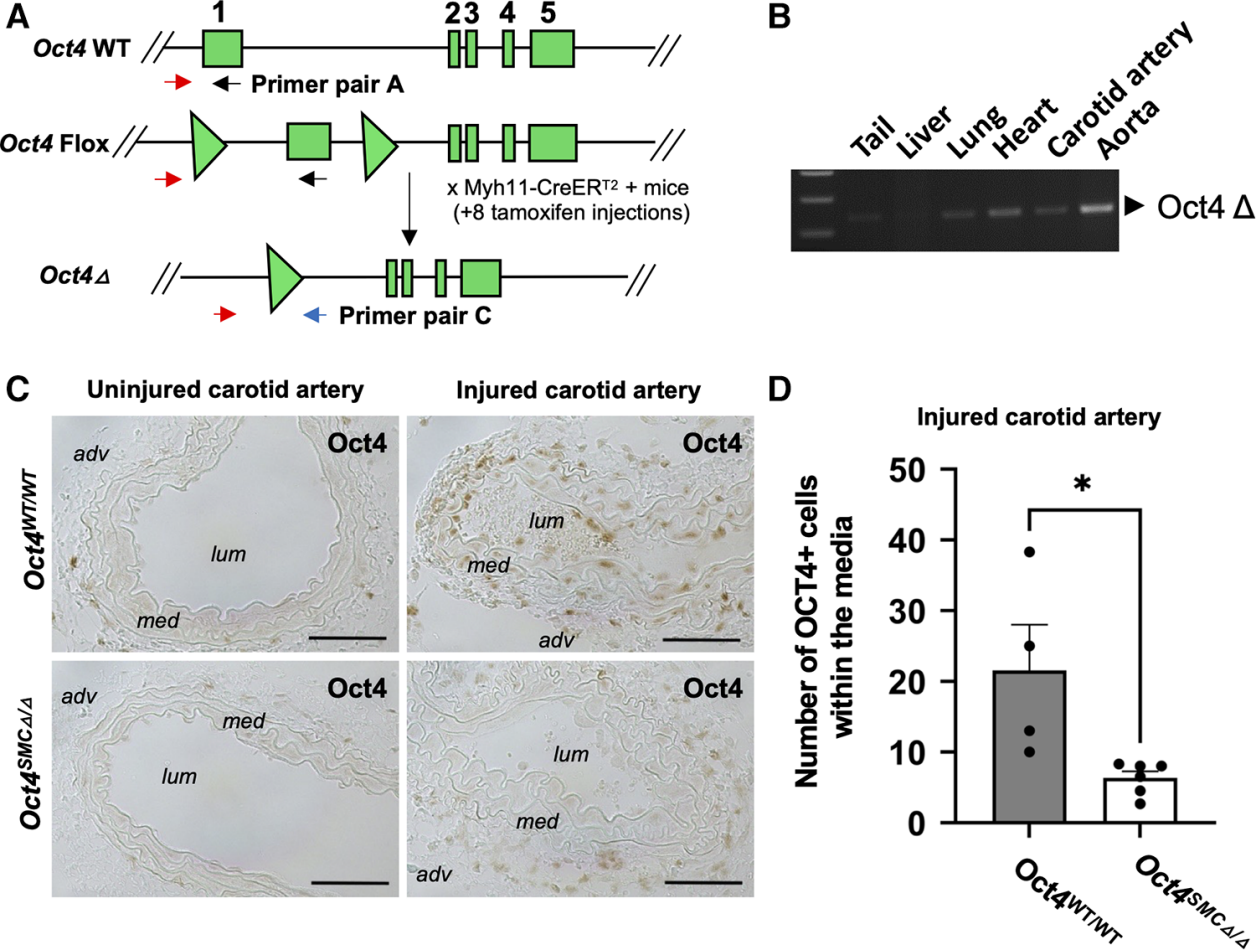
The pluripotency factor OCT4 was reactivated in mouse carotid arteries after carotid ligation injury. (**A**) Schematic of the wild-type (*Oct4* WT), Flox (*Oct4* Flox), and null (*Oct4 Δ*) alleles of the *Oct4* gene. Gene exons 1–5 indicated as rectangles, loxP sites indicated as triangles. Primer set A was used to detect WT/Flox alleles and C to detect Oct4 *Δ* as previously described (5, 26). (**B**) PCR genotyping of tissues from the *Oct4^Δ^*^/*Δ*^*Myh11*-CreERT2 mouse after 8 tamoxifen injections using primer set C, detecting the *Oct4^Δ^* allele. (**C**) OCT4 protein levels were increased in cells within the tunica media of the right injured carotid arteries of *Oct4*^WT/WT^, but not SMC-specific conditional knockout *Oct4^SMCΔ^*^/*Δ*^ mice at day 3 post-carotid ligation injury. Micrographs show cross-sections of injured and uninjured carotid arteries: adv—adventitia, lum—lumen, med—tunica media. Scale bar = 50 µm. (**D**) Quantification of the OCT4 positive medial area. Values represent the mean for three locations ± s.e.m. *P < 0.05 *Oct4*^WT/WT^ (*n* = 4) vs. *Oct4^SMCΔ^*^/*Δ*^ (*n* = 6) mice by non-parametric Mann Whitney test.

*Second*, we used conditional SMC-specific tamoxifen-inducible *Oct4* knockout mice that we previously generated by crossing *Oct4*^Flox/Flox^ mice (26) with mice carrying a tamoxifen-inducible Cre recombinase/estrogen receptor fusion protein under transcriptional control of the *Myh11* promoter (Myh11-CreERT2) (27) (Figure 2A). We previously demonstrated that tamoxifen-treated *Oct4*^Flox/Flox^;*Myh11*-CreERT2 mice showed selective recombination of *Oct4* in SMC-rich tissues such as the aorta (5) and carotid artery (Figure 2B) but no detectable recombination in non-SMC-rich tissues, such as the liver. To determine if OCT4 plays a role in non-atherogenic vascular pathogenesis, SMC-specific Oct4 knockout mice (designated as *Oct4*^SMC*Δ*/*Δ*^) and wild-type mice (designated as *Oct4*^WT/WT^) were subjected to carotid ligation injury. Results of OCT4 immunostaining showed marked induction of the OCT4 expression three days following carotid ligation in *Oct4*^WT/WT^ injured carotid arteries but not in uninjured arteries (Figure 2C). This induction was not observed within medial cells of *Oct4*^SMC*Δ*/*Δ*^ injured carotid arteries (Figure 2D), consistent with SMC-specific gene targeting.

These results provide clear evidence that OCT4 is quickly activated in response to acute vascular injury within the medial SMC, suggesting that this factor has a functional role in vascular remodeling after the injury.

#### SMC-specific loss of OCT4 accelerates SMC proliferation and alters neointima formation following vascular injury

**Figure 3:**
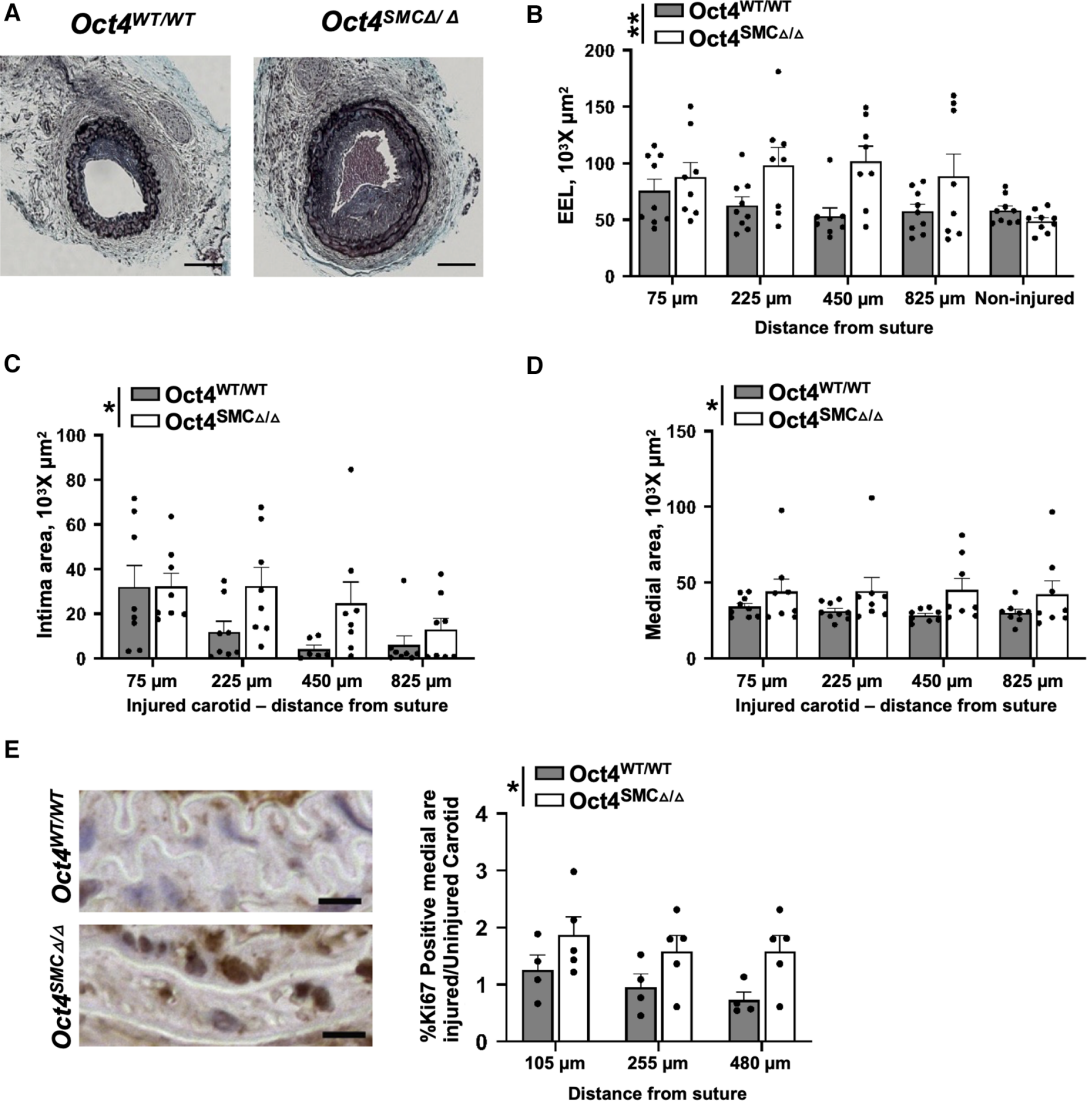
SMC-specific knockout of *Oct4* accelerated neointima formation and increased tunica media following carotid artery ligation injury. (**A**) Movat staining of representative injured carotid arteries of *Oct4*^WT/WT^ and *Oct4^SMCΔ^*^/*Δ*^ mice at day 21 post-carotid ligation injury. Scale bar = 50 µm. (**B**) Total vessel area within external elastic lamina (EEL), (**C**) intimal area (neointima), and (**D**) medial area. Number of injured mice used for analyses: *Oct4*^WT/WT^ (*n* = 8) vs. *Oct4^SMCΔ^*^/*Δ*^ (*n* = 9). (**E**) Ki67 staining of the representative carotid arteries of *Oct4*^WT/WT^ and *Oct4^SMCΔ^*^/*Δ*^ mice at day 3 post-carotid ligation injury. Scale bar = 10 µm. (**F**) Quantification of the Ki67 positive medial area of the ligated (right; Rc) vs. control unligated (left; Lc) carotid artery. Number of mice used for analyses: *Oct4*^WT/WT^ (*n* = 4) vs. *Oct4^SMCΔ^*^/*Δ*^ (*n* = 5). (**B–D,F**) Values represent mean ± s.e.m. *P < 0.05, **P < 0.01 by 2-way ANOVA (**B–D**) or non-parametric ANOVA (**F**) *Oct4*^WT/WT^ vs. *Oct4^SMCΔ^*^/*Δ*^ mice across multiple locations from the ligation suture.

The morphometric analysis of the carotid arteries at 21 days post-injury revealed that genetic inactivation of OCT4 in SMC resulted in significant increases in intimal, medial, and total vessel area of the injured carotid artery injury, as well as outward remodeling as indicated by an increased external elastic lamina (EEL) area (Figures 3A–D). No differences were observed between *Oct4*^SMC*Δ*/*Δ*^ and *Oct4*^WT/WT^ uninjured carotid arteries. Importantly, medial areas of *Oct4*^SMC*Δ*/*Δ*^ injured arteries were increased by 30%–40% compared to controls, suggesting that OCT4 may regulate SMC proliferation. To test this possibility, we stained the injured carotid arteries from *Oct4*^SMC*Δ*/*Δ*^ and *Oct4*^WT/WT^ mice for the proliferation marker Ki67. At 3 days post-injury, Ki67 staining was significantly increased in the injured carotid arteries of *Oct4*^SMC*Δ*/*Δ*^ vs. *Oct4*^WT/WT^ mice (Figures 3E,F). Overall, results indicate that OCT4 plays a critical role in vascular remodeling after injury, at least in part by inhibiting hyper-proliferation of medial SMC. However, we cannot confidently answer the question of whether the increase in medial thickness is due to SMC hypertrophy and/or proliferation since we showed years ago that both involve increased DNA synthesis as hypertrophic SMC become polyploid (32).

#### OCT4 is critical for maintaining SMC contractile state

**Figure 4:**
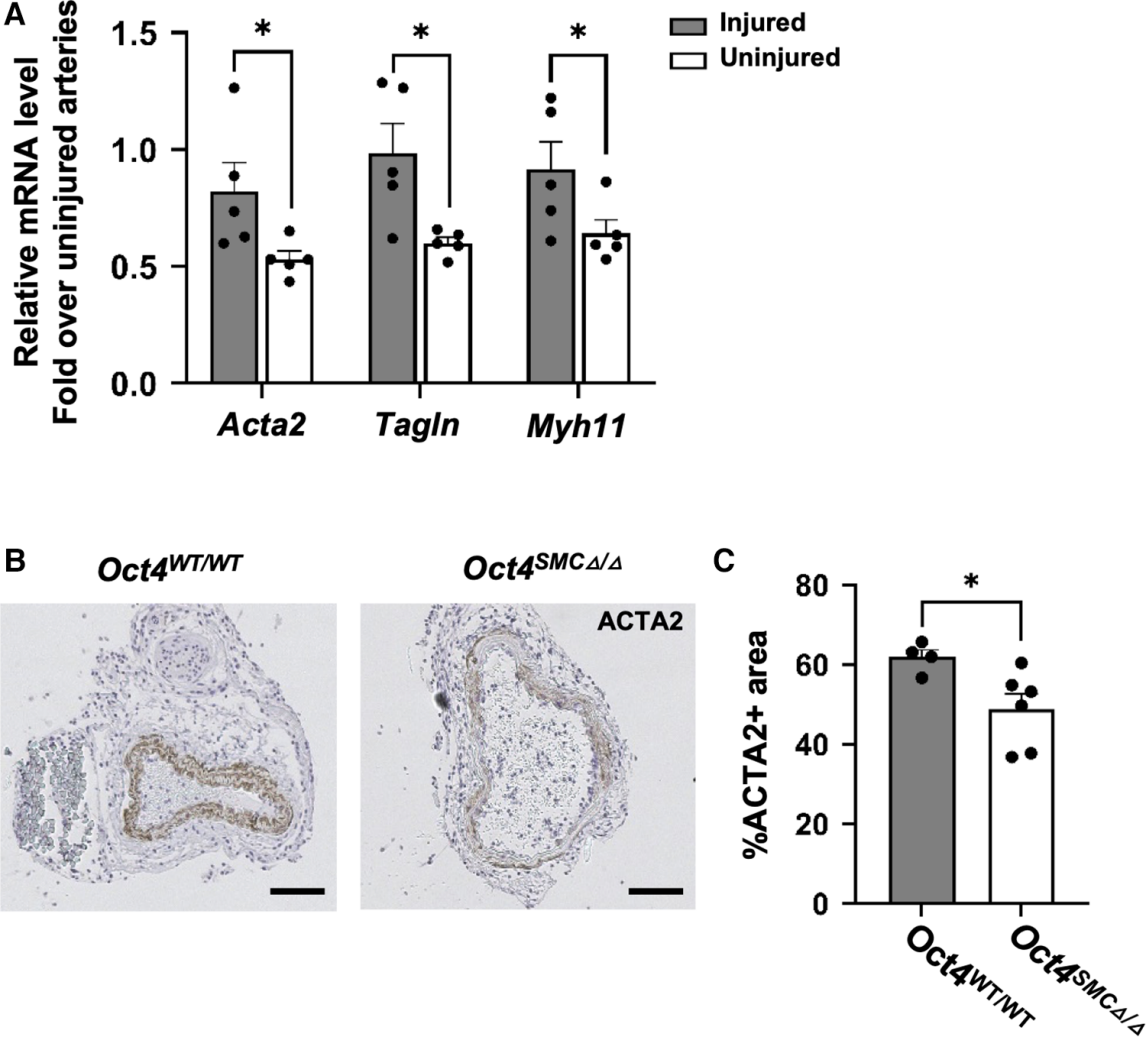
SMC-specific knockout of *Oct4* attenuated SMC phenotypic transition within carotid arteries following carotid ligation injury. (**A**) Total mRNA was isolated from right injured and left uninjured carotid arteries of *Oct4*^WT/WT^ and *Oct4^SMCΔ^*^/*Δ*^ mice 24 h post-injury. Endogenous mRNA levels of *Acta2*, *Tagln*, and *Myh11* were measured by qRT-PCR. For each animal, results for the right injured carotid artery and left uninjured carotid artery were normalized to the result for uninjured animals. *P < 0.05, *Oct4*^WT/WT^ vs. *Oct4^SMCΔ^*^/*Δ*^ (*n* = 5) by Student *t*-test. (**B**) ACTA2 staining of the representative right injured carotid arteries of *Oct4*^WT/WT^ and *Oct4^SMCΔ^*^/*Δ*^ mice at day 3 post-carotid ligation injury. Scale bar = 50 µm. (**C**) Quantification of the ACTA2 positive medial area. Values represent the mean for two locations ± s.e.m. *P < 0.05, *Oct4*^WT/WT^ vs. *Oct4^SMCΔ^*^/*Δ*^ mice by Student *t*-test.

Given that SMC proliferation is a characteristic of dedifferentiated SMC, we examined if *Oct4* is critical for maintaining the contractile state of SMC. We found that OCT4 deficiency accelerated downregulation of SMC contractile gene expression, in that *Oct4*^SMC*Δ*/*Δ*^ injured carotid arteries demonstrated lower expression of SMC contractile genes *Acta2* and *Tagln* compared to *Oct4*^WT/WT^ 24 h post-injury (Figure 4A). In addition, immunohistochemistry staining for ACTA2 in injured carotid arteries on day 3 post-injury confirmed that loss of OCT4 in SMC accelerates suppression of this contractile protein within the tunica media (Figures 4B,C).

**Figure 5:**
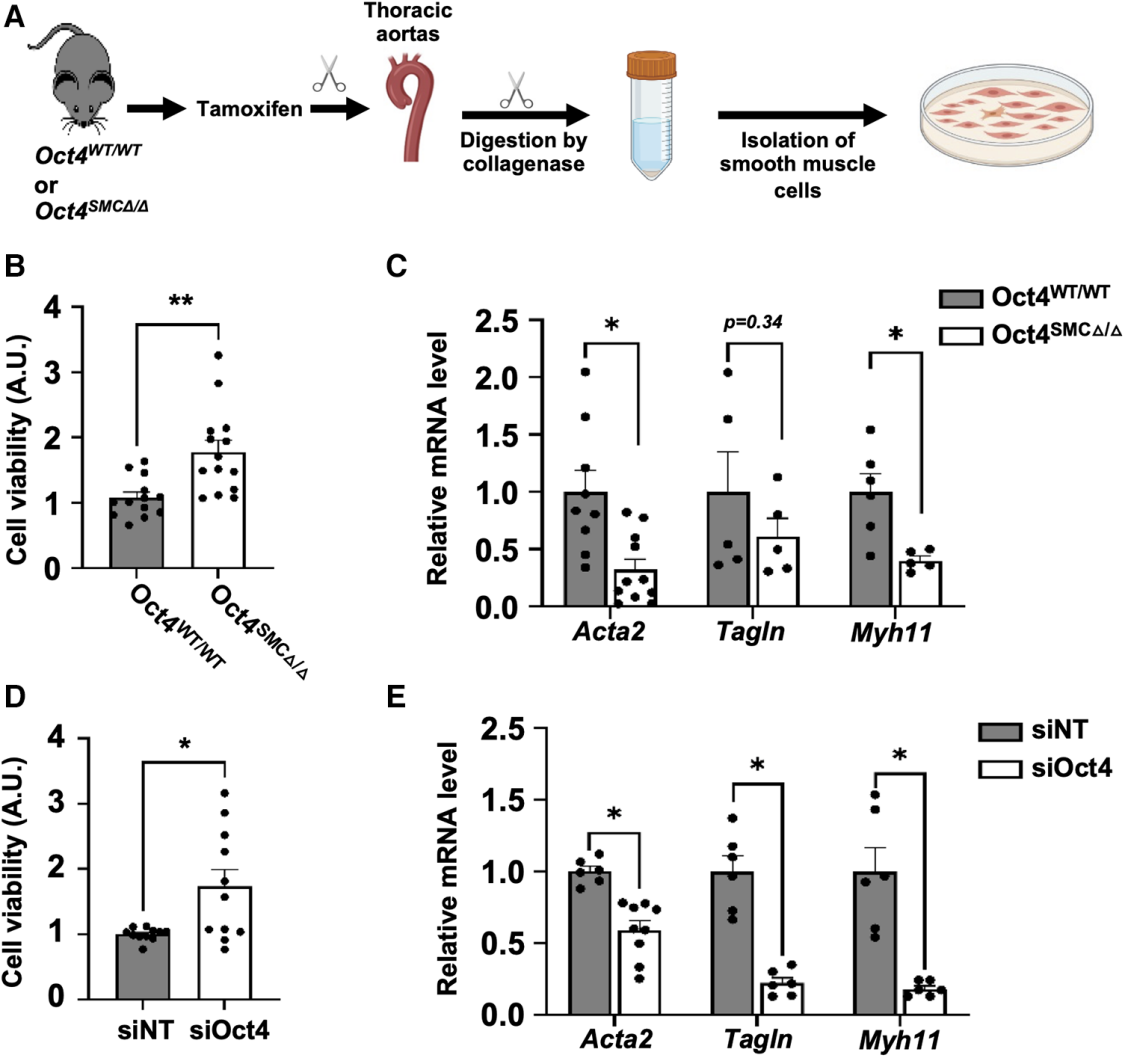
Loss of OCT4 attenuated SMC phenotypic transition in cultured SMC. (**A**) SMC were isolated from thoracic aortas of *Oct4*^WT/WT^ or *Oct4^SMCΔ^*^/*Δ*^ mice using the enzymatic method. For all *in vitro* experiments, cells were cultured in serum-free media for 48 h before the experiment to differentiate and synchronize SMC. After starvation, Oct4 wild-type (Oct4 WT) and Oct4 knockout (Oct4 KO) (**B,C**) or wild-type SMC transfected with si*Oct4* or non-target siRNA (siNT) (**D,E**) were grown in culture media containing 2% serum. (**B,D**) Relative viable cell numbers were counted after 24 h based on the absorbance at 485 nm using a CellTiter 96® AQueous One Solution Cell Proliferation assay (MTS assay). Data represent mean ± s.e.m. *P < 0.05, **P < 0.01 by 2 way-ANOVA; *n* = 12−15 replicates from 3 experiments. (**C,E**) Endogenous mRNA levels of *Acta2*, *Tagln*, and *Myh11* were measured by qRT-PCR. Data represent mean ± s.e.m. *P < 0.05, **P < 0.01 by 2 way-ANOVA; *n* = 6–9 replicates from 3 experiments.

To further investigate the mechanisms whereby OCT4 regulates contractile SMC, SMC were isolated from the aortas of *Oct4*^SMC*Δ*/*Δ*^ vs. *Oct4*^WT/WT^ mice one week after the last tamoxifen injection (Figure 5A). We previously demonstrated that SMC from *Oct4*^SMC*Δ*/*Δ*^ mice (*Oct4* KO) have ∼96% of Flox locus recombination (5). O*ct4* KO SMC demonstrated increased growth rates (Figure 5B) compared to *Oct4* wild-type SMC (*Oct4* WT). Quantitative RT-PCR analysis showed that *Oct4* KO SMC had lower levels of SMC contractile genes, *Acta2*, *Tagln*, and *Myh11* (Figure 5C) compared to *Oct4* WT SMC. Importantly, the knockdown of *Oct4* using blocking siRNA resulted in similar increases in cell proliferation and suppression of SMC contractile genes (Figures 5D,E), indicating that these changes are not a result of the different adaptation of *Oct4* KO SMC to the cell culture condition.

**Figure 6:**
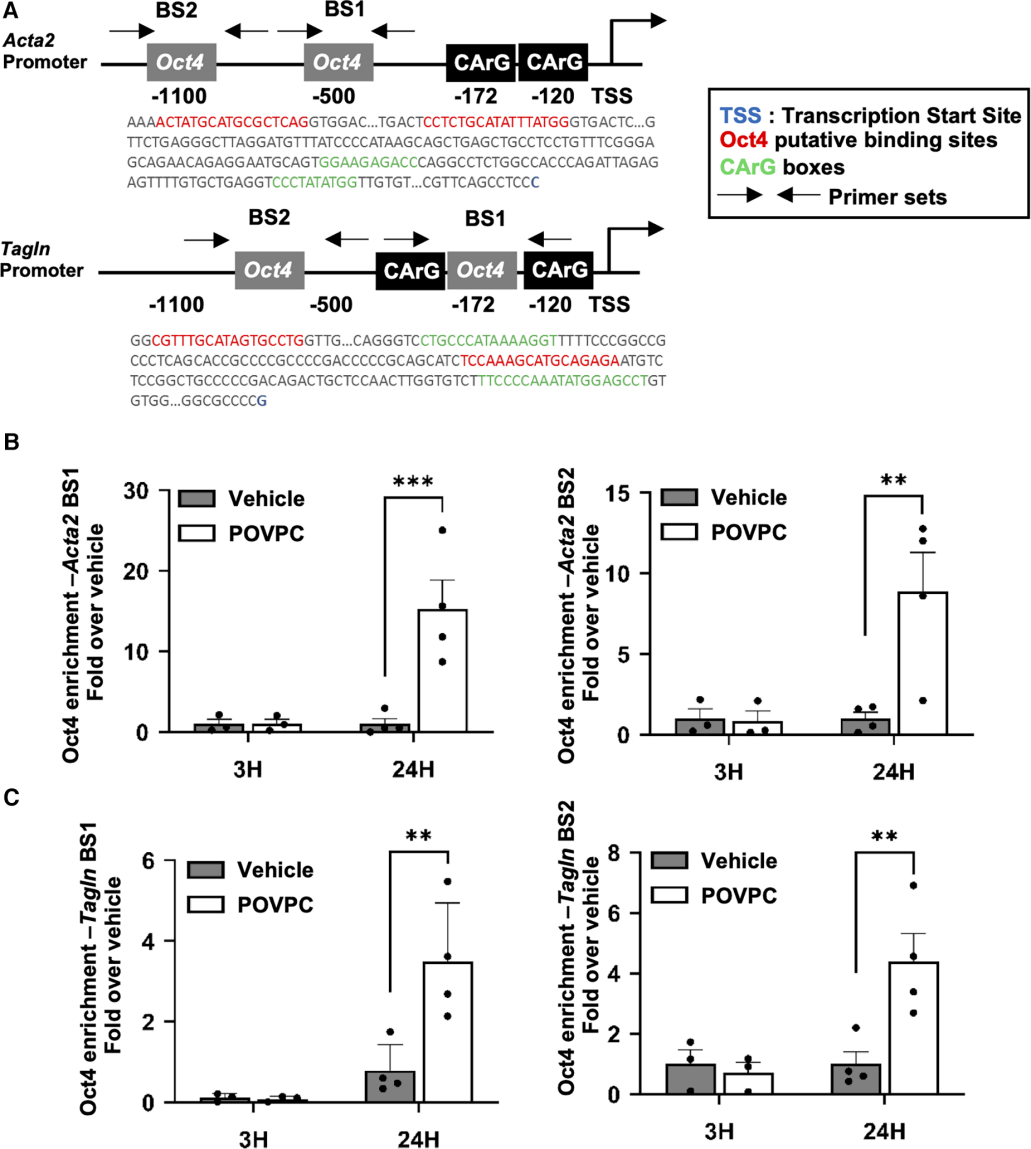
OCT4 regulates the expression of *Acta2* and *Tagln* via direct binding to the gene promoter elements. (**A**) Schematic representation of the *Acta2* and *Tagln* promoter regions. BS—binding site. (**B,C**) Mouse aortic SMC were treated with either POVPC (10 µg/mL) or DMSO-vehicle for 3 or 12 h. The enrichment of OCT4 on the promoter region of the *Acta2* (**B**) or *Tagln* (**C**) genes was determined by ChIP assays. Chromatin precipitated with antibody for OCT4 was subjected to the qRT-PCR. Results were normalized to the total Input and demonstrated as a fold-increased over vehicle. *P < 0.05 POVPC (10 µg/ml) vs. Vehicle control by Student's *t*-test.

Then, we conducted further mechanistic studies based on the observation that both the *Acta2* and *Tagln* proximal promoters contain conserved binding sites for OCT4 (Figure 6A). We have recently demonstrated that the pro-atherogenic phospholipid POVPC [1-palmitoyl-2-(oxovaleroyl)-*sn*-glycero-3-phosphocholine] is a potent repressor of SMC marker gene expression in vascular SMC, as well as in rat and mouse carotid arteries *in vivo* (8, 33). We also demonstrated that POVPC induces migration of cultured SMC, at least in part via OCT4 activation (33). Here, we found that treating mouse aortic SMC with POVPC was associated with a significant enrichment of OCT4 on the *Acta2* and *Tagln* promoters (Figures 6B,C).

**Figure 7:**
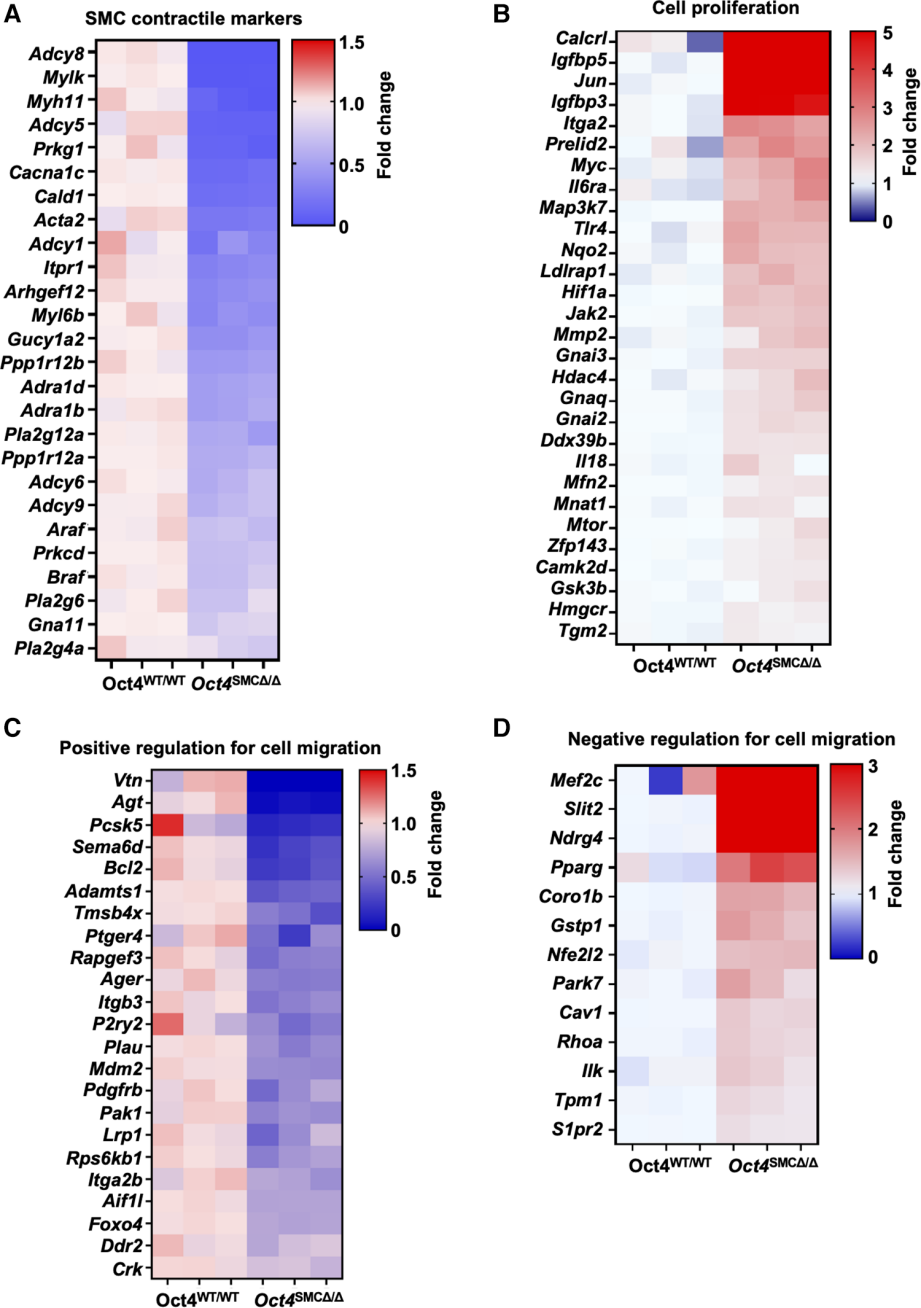
RNAseq analysis revealed significant dysregulation of genes related to SMC contraction, proliferation, and migration in OCT4 deficient SMC. Bulk RNAseq was performed on cultured mouse aortic *Oct4*^WT/WT^ and *Oct4^SMCΔ^*^/*Δ*^ treated with the oxidized phospholipid POVPC (10 µg/ml) for 24 h. The heat maps show the differential gene expression related to SMC contraction (**A**), cell proliferation (**B**), and positive and negative regulation of cell migration (**C**,**D**).

Finally, to further assess OCT4-dependent gene profiles, we analyzed our previously published RNAseq in cultured *Oct4* KO vs. WT SMC (GSE75044). Our differential expression (DE) analysis demonstrated the marked dysregulation of genes related to the SMC contraction, including downregulation of contractile genes *Adcy8, Myh11, Mylk, Cald1, Acta2, and Myl6b* [GO:0045987], in *Oct4* KO SMC. (Figure 7A). In addition, our analysis revealed significant up-regulation of genes related to the positive regulation of SMC proliferation [*Igfbp5, Jun, Igfbp3, Itga2, and Myc* (GO:0048661))] in *Oct4* KO cells compared to wild-type cells (Figure 7B). These RNAseq analyses are consistent with the data showing that loss of OCT4 results in hyper-proliferation of SMCs. Also, we previously reported that loss of OCT4 in SMC decreases SMC migration *in vitro* and *ex vivo* (5). Consistently with these decreases in cell migration, RNAseq analyses showed down-regulation of genes belonging to the positive regulation of SMC migration [*Vtn, Agt, Pcsk5, and Bcl2* (GO:0014911)] and up-regulation of genes belonging to the negative regulation of SMC migration [*Mef2c, Slit2, Ndrg4, and Pparg* (GO0014912)] (Figures 7C,D). The molecular mechanisms responsible for the differences in cell migration vs. proliferation are not fully understood. Our RNAseq analyses in *Oct4* knockout and wild-type SMC demonstrate that loss of OCT4 resulted in dysregulation of genes, which might be responsible for the difference in SMC migration vs. proliferation responses. Future studies are needed to further understand this difference.

## Discussion

The pluripotency factor OCT4 is one of the essential transcriptional factors regulating the early stages of embryonic development and one of the critical Yamanaka factors used for iPS cell transformations. Until recently, the dogma was that OCT4 is irreversibly epigenetically silenced in adult cells (34). However, we and others found that OCT4 is reactivated and plays functional role in vascular SMC (35, 36) in pathological conditions, including atherosclerosis, aortic aneurism, and pulmonary hypertension. We also demonstrated that OCT4 plays an important atheroprotective role by regulating SMC migration (5). Results of the current study demonstrate that OCT4 also has a critical role in SMC-dependent blood vessel wall remodeling after acute vascular injury, at least in part by promoting SMC contractile phenotype via direct interaction with SMC contractile genes, ACTA2 and TAGLN, and regulating expression of genes involved in inhibition of SMC proliferation.

We previously demonstrated that another pluripotency transcriptional factor KLF4 is a master regulator of SMC phenotypic switching, including both direct binding to the promoters of the SMC marker genes (28) and indirect effects of KLF4 via down-regulating the expression of the SMC-specific serum response factor (SRF) coactivator myocardin and preventing SRF/myocardin complexes from associating with SMC gene promoters (11, 12). Although KLF4 positively regulates OCT4 in SMC (5), we observed that the genetic inactivation of KLF4 and OCT4 in SMC resulted in opposite effects on atherosclerosis development. Thus, conditional knockout of *Oct4*, specifically in SMC, led to marked increases in lesion size and decreases in multiple indices of plaque stability, including a less formed fibrous cap, larger necrotic core, and increased intra-plaque hemorrhage (5). SMC-lineage tracing studies demonstrated a marked reduction in the numbers of SMC within lesions and the fibrous cap (5). Conversely, SMC-specific knockout of KLF4 resulted in smaller and more stable lesions (29). Importantly, SMC-specific knockout of KLF4 led to a decrease in SMC transition to multiple phenotypes, including LGALS3^+^SPP1^+^ osteogenic-like cells (17). At the same time, previous studies demonstrated that, similarly to OCT4, KLF4 deficiency resulted in hyper-proliferation of SMC *in vitro* and *in vivo* after carotid ligation injury (28) but inhibited migration of SMC *in vitro* (33). Our results herein provide evidence that OCT4 directly promotes differentiation of SMC that is opposite to KLF4, which suppresses SMC differentiation, but further highlights the importance of the pluripotency gene network in controlling SMC phenotypic transitions. Results are consistent with those of *Alencar* et al. (17), showing that SMC-KLF4 and SMC-Oct4 KO mice exhibit virtually opposite transcriptomic signatures. Our new findings raise additional questions about the complexity of these pluripotency factor interactions to modulate SMC plasticity. It is interesting to speculate that the fine-tuning of these OCT4- and KLF4-dependent mechanisms and their crosstalk are responsible for the directivity of the SMC phenotypic transitions. In particular relevance, our *in vivo* ChIPseq analyses on chromatin from mouse atherosclerotic arteries demonstrated that 39% of accepted human coronary artery disease (CAD) genome-wide association study (GWAS) loci (37) are the nearest gene binding targets of KLF4 or OCT4 specifically in SMC (17).

Given the massive change in gene expression in *Oct4* KO SMC compared to wild-type cells, the likely effect of OCT4 on SMC phenotypic transitions is very complex, affecting numerous molecular pathways simultaneously. This suggests that environmental cues (e.g., hypoxia, hyperlipidemia, inflammation, etc.) may contribute to the OCT4-dependent modulation of SMC transitions. For instance, we found that Toll-like receptor 4 (TLR4) was significantly upregulated in *Oct4* KO cells. Therefore, different TLR4 ligands would potentially induce different responses in SMC.

Importantly, we previously found that the lentivirus-driven overexpression of OCT4 resulted in increased migration and decreased proliferation of SMC (5). Moreover, we demonstrated that the knockdown of *Oct4* in SMC precursor cells (A404 (38), expressing a high level of OCT4 and differentiating toward contractile SMC in response to all trans retinoic acid (at-RA), was sufficient to initiate the SMC differentiation program in these cells, including up-regulation of *Acta2* and *Tagln* (5). Although these “high OCT4 expression” experiments recapitulate the iPSC/stem cell state, they suggest that OCT4 plays a fundamental role in SMC not only in pathological conditions but also in SMC differentiation during embryonic development.

In conclusion, our current study provides evidence that the SMC-derived pluripotency factor OCT4 is critical for vascular remodeling in response to injury by regulating SMC contractility and proliferation. Future studies are needed to further elucidate endogenous OCT4-dependent upstream and downstream mechanisms of SMC phenotypic transitions.

## Data Availability

The datasets presented in this study can be found in online repositories. The names of the repository/repositories and accession number(s) can be found in the article/**Supplementary Material**.
