# Supplementary material for: Smooth muscle cells-specific loss of OCT4 accelerates neointima formation after acute vascular injury
Source: Front Cardiovasc Med. 2023 Oct 23;10:1276945. doi: 10.3389/fcvm.2023.1276945 (PMC10627795; doi:10.3389/fcvm.2023.1276945)
Supplement: Supplementary file 1 [file Datasheet1.pdf]

*Shin et al., Supplementary Figure 1*

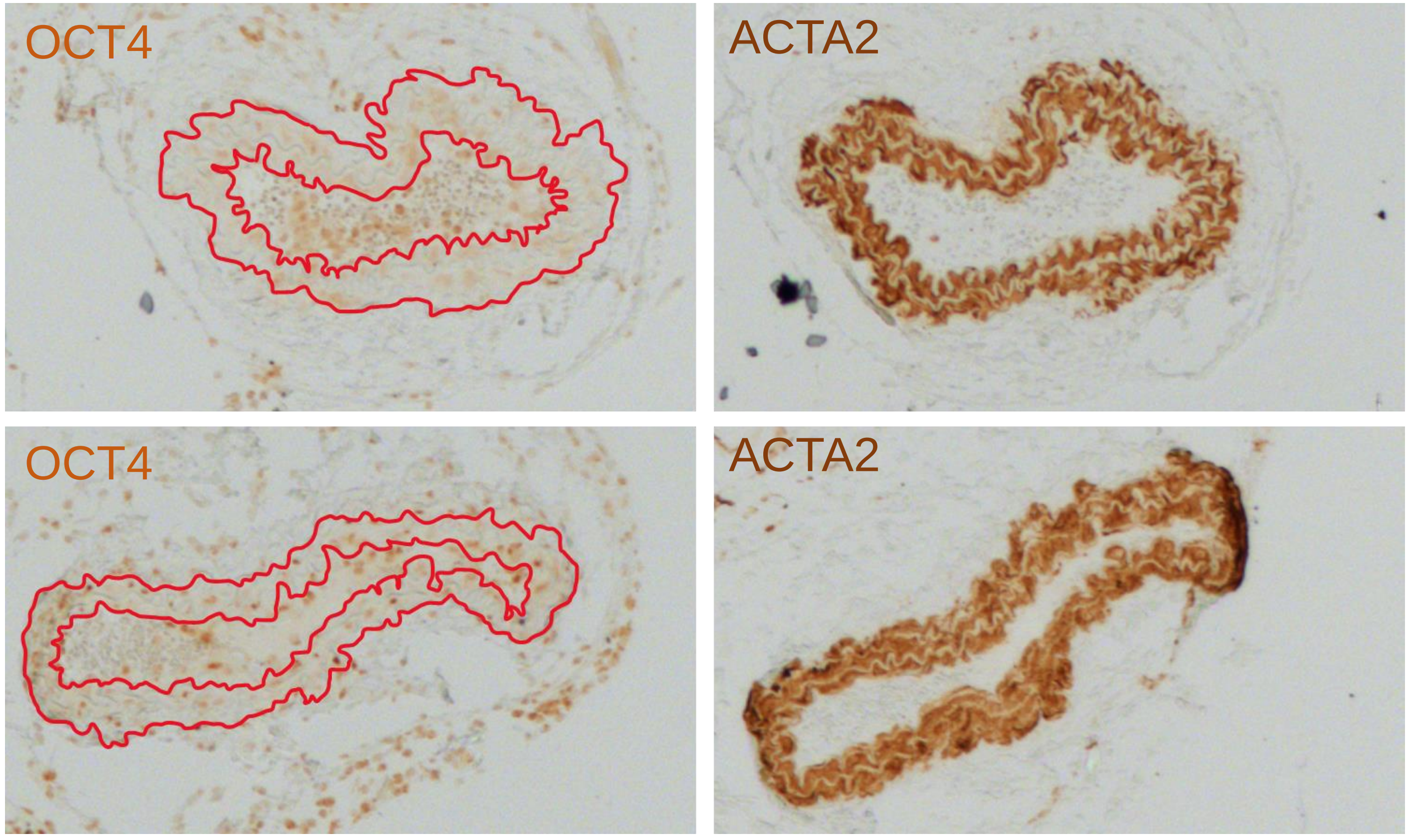


**Supplementary Figure 1.** Representative immunohistochemistry staining for OCT4 and ACTA2 on the sequential sections (step +5 µm). For OCT4 signal quantification, tunica media was outlined following internal and external elastic lamina (red lines). For each carotid artery, 3 locations (70 µm, 220 µm, and 445 µm from the suture) were stained. As no statistical difference was noted between different locations, results were averaged for each carotid artery.
